# Effects of a hard stop for *C. difficile* testing: Provider uptake and patient outcomes

**DOI:** 10.1017/ash.2023.280

**Published:** 2023-09-29

**Authors:** Danielle Doughman, David Weber, Nikolaos Mavrogiorgos, Shelley Summerlin-Long, Michael Swartwood, Alexander Commanday, Lisa Stancill, Nicholas Kane, Emily Sickbert-Bennett Vavalle

## Abstract

**Background:** *Clostridioides difficile* infection (CDI) is a serious healthcare-associated infection responsible for >12,000 US deaths annually. Overtesting can lead to antibiotic overuse and potential patient harm when patients are colonized with *C. difficile*, but not infected, yet treated. National guidelines recommend when testing is appropriate; occasionally, guideline-noncompliant testing (GNCT) may be warranted. A multidisciplinary group at UNC Medical Center (UNCMC) including the antimicrobial stewardship program (ASP) used a best-practice alert in 2020 to improve diagnostic stewardship, to no effect. Evidence supports use of hard stops for this purpose, though less is known about provider acceptance. **Methods:** Beginning in May 2022, UNCMC implemented a hard stop in its electronic medical record system (EMR) for *C. difficile* GNCT orders, with exceptions to be approved by an ASP attending physician. Requests were retrospectively reviewed May–November 2022 to monitor for adverse patient outcomes and provider hard-stop compliance. The team exported data from the EMR (Epic Systems) and generated descriptive statistics in Microsoft Excel. **Results:** There were 85 GNCT orders during the study period. Most tests (62%) were reviewed by the ASP, and 38% sought non-ASP or no approval. Of the tests reviewed by the ASP, 33 (62%) were approved and 20 (38%) were not. Among tests not approved by the ASP, no patients subsequently received CDI-directed antibiotics, and 1 patient (5%) warranted same-admission CDI testing (negative). Of tests that circumvented ASP review, 18 (56%) ordering providers received a follow-up email from an associate chief medical officer to determine the rationale. No single response type dominated: 3 (17%) were unaware of the ASP review requirement, 2 (11%) indicated their patient’s uncharted refusal of laxatives, 2 (11%) indicated another patient-specific reason. Provider avoidance of the ASP approval mechanism decreased 38%, from 53% of noncompliant tests in month 1 to 33% of tests in month 6. Total tests orders dropped 15.5% from 1,129 during the same period in 2021 to 954 during the study period (95% CI, 13.4%–17.7%). Compliance with the guideline component requiring at least a 48-hour laxative-free interval prior to CDI testing increased from 85% (95% CI, 83%–87%) to 95% (95% CI, 93%–96%). CDI incidence rates decreased from 0.52 per 1,000 patient days (95% CI, 0.41–0.65) to 0.41 (95% CI, 0.32–0.53), though the change was neither significant at *P* = .05 nor attributable to any 1 intervention. **Conclusions:** Over time and with feedback to providers circumventing the exception process, providers accepted and used the hard stop, improving diagnostic stewardship and avoiding unneeded treatment.

**Disclosures:** None

